# Effects of Vestibular Rehabilitation Interventions in the Elderly with Chronic Unilateral Vestibular Hypofunction

**Published:** 2017-07

**Authors:** Arash Bayat, Nader Saki

**Affiliations:** 1 *Musculoskletal Rehabilitation Research Center, Ahvaz Jundishapur University of Medical Sciences, Ahvaz, Iran.*; 2 *Hearing Research Center, Ahvaz Jundishapur University of Medical Sciences, Ahvaz, Iran.*

**Keywords:** Vestibular, Rehabilitation, Elderly

## Abstract

**Introduction:**

Although vestibular rehabilitation therapy (VRT) methods are relatively popular in treating patients with body balance deficits of vestibular origin, only limited studies have been conducted into customized exercises for unilateral vestibular hypofunction (UVH). Furthermore, very little evidence is available on the outcomes of VRT in the elderly population with chronic UVH.

**Materials and Methods:**

A total of 21 patients, aged 61 to 74 years, with UVH participated in this study. The dizziness handicap inventory (DHI) was performed immediately before, and 2 and 8 weeks after treatment.

**Results:**

All patients showed a reduction in DHI scores during the study. The average decrease in DHI score was 25.98 points after 2 weeks’ intervention (P<0.001) and 32.54 points at the end of the study. This improvement was observed in all DHI subscores, and was most profound in the functional aspect. The correlation between the degree of final recovery and canal paresis was not significant (P>0.05). There were no relationships between the scores and gender.

**Conclusion:**

Our study demonstrates that VRT is an effective method for the management of elderly patients with UVH, and shows maximal effect on functional aspects.

## Introduction

Dizziness is a common complaint among the elderly. The prevalence of vestibular disorder increases with age, affecting around 70% of individuals attending geriatric outpatient settings (1-3). Dizziness in the elderly is considered to originate from physiological alterations in the peripheral and central structures of the vestibular system. The aging process gradually compromises vestibular end organ receptors, leading to a significant reduction in vestibular nerve conduction velocity (3–6). These changes manifest as disequilibrium, falls, anxiety and loss of confidence (7,8).

Unilateral vestibular hypofunction (UVH) is a disorder that creates a reduced total or partial reduction in vestibular function on one side of the body (9). Patients with UVH frequently report symptoms such as dizziness, oscillopsia, postural instability, and gait disorders (9–12). These impairments produce significant limitations in activity and participation in the affected patient (10,13).

Surgical or pharmacologic procedures offer only limited improvement in cases of chronic vestibular dysfunction. Therefore, vestibular rehabilitation therapy (VRT) has attracted increased interest in the management of patients experiencing chronic UVH (14–16). VRT exercises are designed to facilitate central nervous system plasticity by generating substitution, habituation, and adaptation mechanisms to enhance postural stability in disorders which lead to conflicting sensory information (17,18). It has been demonstrated that VRT methods have a positive effect in improving gait and quality of life, and in reducing the symptoms of dizziness, depression, and anxiety (19–21).

Although VRT is relatively popular, only limited studies have been utilized investigating customized exercises for unilateral vestibular dysfunction. Furthermore, very little evidence is available on the outcomes of VRT in elderly patients with UVH. The objective of the current study was to assess the effect of VRT on chronic UVH in the elderly population.

## Materials and Methods

*I. Participants*

This study was initiated after approval of the institutional ethics committee for human subject research. All subjects had verified vertigo and were reviewed by a panel of consultant specialists including a neurologist, otologist, and audiologist. As a result, 21 patients (61 to 74 years of age) diagnosed by our team with chronic decompensated unilateral vestibular deficit participated in the study. Patients with visual, cervical, or neurologic involvement were excluded. Those with fluctuating vertigo, a symptom duration of <2 months, and a history of rehabilitation in the last 4 months were also excluded.

*II. Examinations*

All subjects underwent full neurotologic assessments, including spontaneous nystagmus, saccades, gaze, tracking, and balance function tests. Unilateral vestibular dysfunction was confirmed by both sideway stepping test (SST) and caloric assessments. Both tests were conducted by blinded examiners.

- **SST test: **Subjects were asked to stand with their feet together and their hands by their sides, and were asked to close their eyes. The SST was considered positive if there was any lean to one side or, in some cases, forward or backward (22).
- **Caloric assessment:** Subjects were evaluated with an air caloric test at 50°C and 24°C (ICS Chartr 200, GN Otometrics, Denmark). UVH was diagnosed according to the criterion of >25% difference in slow-phase velocity between the two ears (23,24).

*III. Treatment program*

According to the case history, physical evaluation, and vestibular tests for each patient, an exercise plan was developed. During the physical examination, the musculoskeletal system (e.g., coordination and proprioception), and oculomotor functions (e.g., tracking, saccadic eye movements, and head thrust) were evaluated. This study was a single-blinded controlled trial, and all assessments were recorded by the same blinded examiner. The treatment protocol consisted of a combination of vestibular adaptation and habituation exercises.

- **Adaptation exercises:**

To improve gain of the vestibular responses to head movements (25,26), patients were asked to stabilize their gaze onto a target during rapid head motions, while maintaining the target in focus (F1 point). On progression to F2 point, the target point and the patient’s head rotated in equal and opposite yaw directions.

- **Habituation** **exercises:**

These exercises were carried out to reduce symptoms and pathological responses produced by repetitive exposure to the provoking stimulus (27). Habituation exercises were performed based on the Brandt and Daroff method (28,29).

The therapy was performed by two experienced audiologists and consisted of an 8-week course of rehabilitation sessions. Each session included a series of 25- to 40- minute exercises, four times a week. The Persian version of the dizziness handicap inventory (DHI) was used to measure the self-perceived handicapping effects of dizziness/ vertigo (30). The inventory contains 25 items that evaluate a respondent’s performance along physical, emotional, and functional dimensions (31). Each item provides a choice of three responses: no (zero point), sometimes (two points), or yes (four points). The total score ranges from 0 to 100, where higher scores imply greater level of disability. Scores of 0-231,31-60,and61-100 represent mild,moderate ,and severe dizziness, respectively (32).

*IV. Statistical analysis*

SPSSv.18 was utilized for statistical analysis. The Wilcoxon signed rank sum test was used to compare DHI scores obtained before and after intervention. Pre-VRT and post-VRT differences between genders were analyzed using the Kruskal–Wallis test. The statistical significance level was established at P<0.05.

## Results

Twenty-one patients (11 males and 10 females, mean age 67.71±5.43 years) participated in this study. Oculomotor function tests (gaze, saccades, and smooth pursuit) were within normal limits in all patients. All subjects showed a reduction in DHI scores. At the initial visit, 71% (15/21) of patients experienced moderate or severe impairment (Table 1). However, at the end of the intervention, this value decreased to 23% (P<0.001).

The mean component and total DHI scores are given in Table 2. The average decrease in DHI score was 25.98 points after 2 weeks of intervention (P<0.001), and 32.54 points at the end of the entire intervention. For all DHI components (emotional, functional, and physical), these differences were found to be statistically significant (P<0.05). The difference was most noticeable in terms of the functional aspect.

No statistical difference was found between gender and total DHI improvement score (P>0.05). The relationship between the degree of final recovery (total DHI score) and canal paresis was not meaningful (P>0.05).

**Table 1:** Comparison of severity of disability (DHI) before and after the exercise program

**Severity of Disability**	**Before intervention**	**After intervention**	**p- value**
Mild	3	16	P<0.001
Moderate	8	3	
Severe	10	2	

**Table 2:** Comparison of the average of DHI score during different stage of the study

**Ear**	**Before intervention**		**2 weeks intervention**		**8 weeks intervention**		**p-value**
	**Mean**	**SD**	**Mean**	**SD**	**Mean**	**SD**	
DHI-physical	15.14	7.43	8.34	9.11	6.03	4.79	P<0.001
DHI-emotional	13.52	6.68	4.85	5.67	3.56	2.44	P=0.008
DHI-functional	20.77	11.67	10.71	8.49	6.75	5.81	P<0.001
DHI-total	51.07	21.22	23.90	16.57	16.34	12.49	P<0.001

## Discussion

The current investigation indicates that customized VRT exercises decrease symptoms and improve dizziness-related disability in the elderly population. While the majority of patients showed high levels of disability at the beginning of the study, most improved to a mild level of impairment by the end of the intervention.

This study shows that after an exercise program lasting 2 weeks, a rapid recovery was achieved. We also found a remarkable difference in total DHI scores between the second week and the end of the treatment program. However, this improvement was most evident after the first 2 weeks of exercises, suggesting that the rehabilitation programs had their maximum influence during the first 2 weeks.

Our findings demonstrate that participants reported lower scores on the emotional component, with more than half of the subjects reporting mild emotional difficulty. In contrast, physical and functional problems were more often moderate or severe. These results are in accordance with findings reported by Voorde et al. and Vereeck et al. Further, Lin et al. found that a functional complaint was most prevalent aspect of dizziness-induced disability (33-35), and also reported that the emotional impairment constituted the least affected aspect.

The aging process gradually causes anatomical and neurophysiological changes within the vestibular system, resulting in vertigo and dizziness. It has been shown that the vestibular sensory epithelia undergo alterations in subjects over the age of 70 years (36,37). These structural changes would be expected to result in diminished balance performance and greater problems in recovering from a vestibular deficit. When the peripheral vestibular system is damaged unilaterally, neuronal activity reaching the ipsilateral vestibular nuclei is diminished compared with that arriving on the contralateral side. Thus, the brain interprets the asymmetry between resting firing rates as a head rotation toward the contralateral ear. This may result in disorientation, oscillopsia, or postural instability (38–40).

A reduction in the severity of dizziness-induced handicap with VRT provides patients with independence in activities of daily living and improves their quality of life. However, the improvement in disability among our participants was not similar, suggesting that disability in the elderly population is a complex phenomenon and may be explained by differences in attitudes to dizziness and coping strategies (41–43).

Unlike Bamiou et al. (44), who reported that dizziness disability is higher in subjects with severe unilateral deficit than in those with a milder condition, we did not observe any association between final recovery and degree of vestibular weakness.

## Conclusion

The results of the current study suggest that vestibular rehabilitation has a positive influence on symptoms in the elderly with chronic UVH. Such exercises lead to an improvement in balance and postural stability, and a decrement in self-reported measure of handicap.
